# Metagenomic and machine learning-aided identification of biomarkers driving distinctive Cd accumulation features in the root-associated microbiome of two rice cultivars

**DOI:** 10.1038/s43705-023-00213-z

**Published:** 2023-02-22

**Authors:** Zhongyi Cheng, Qiang Zheng, Jiachun Shi, Yan He, Xueling Yang, Xiaowei Huang, Laosheng Wu, Jianming Xu

**Affiliations:** 1 Institute of Soil and Water Resources and Environmental Science, College of Environmental and Resource Sciences, Zhejiang University, Hangzhou, 310058 China; 2 Zhejiang Provincial Key Laboratory of Agricultural Resources and Environment, Hangzhou, 310058 China; 3 Department of Mathematics and Theories, Peng Cheng Laboratory, Shenzhen, 518000 China; 4 Department of Environmental Sciences, University of California, Riverside, CA 92521 USA

**Keywords:** Environmental sciences, Soil microbiology

## Abstract

Developing low-cadmium (Cd) rice cultivars has emerged as a promising avenue for food safety in Cd-contaminated farmlands. The root-associated microbiomes of rice have been shown to enhance rice growth and alleviate Cd stress. However, the microbial taxon-specific Cd resistance mechanisms underlying different Cd accumulation characteristics between different rice cultivars remain largely unknown. This study compared low-Cd cultivar XS14 and hybrid rice cultivar YY17 for Cd accumulation with five soil amendments. The results showed that XS14 was characterized by more variable community structures and stable co-occurrence networks in the soil-root continuum compared to YY17. The stronger stochastic processes in assembly of the XS14 (~25%) rhizosphere community than that of YY17 (~12%) suggested XS14 may have higher resistance to changes in soil properties. Microbial co-occurrence networks and machine learning models jointly identified keystone indicator microbiota, such as *Desulfobacteria* in XS14 and *Nitrospiraceae* in YY17. Meanwhile, genes involved in sulfur cycling and nitrogen cycling were observed among the root-associated microbiome of these two cultivars, respectively. Microbiomes in the rhizosphere and root of XS14 showed a higher diversity in functioning, with the significant enrichment of functional genes related to amino acid and carbohydrate transport and metabolism, and sulfur cycling. Our findings revealed differences and similarities in the microbial communities associated with two rice cultivars, as well as bacterial biomarkers predictive of Cd-accumulation capacity. Thus, we provide new insights into taxon-specific recruitment strategies of two rice cultivars under Cd stress and highlight the utility of biomarkers in offering clues for enhancing crop resilience to Cd stresses in the future.

## Introduction

Understanding the characteristics of the root-associated microbiome is an emergent research priority; such information is critically vital for us to comprehend the profound roles of microbiomes in promoting crop growth and tolerance under environmental stress [1–3]. Plant root-associated microbial community compositions and functions are largely differentiated by root microhabitats and phenotypes [4–6]. Recent studies have reported niche-specific recruitment and function-specific microbial enrichment that were triggered by plant root microbiomes’ responses to environmental perturbation [7–9]. Additionally, crop hosts have selection effects on microbiomes in root-associated niches that could induce the restructured community structure and transformation of functional profiles [10, 11]. Despite the growing recognition that root-associated microbiome community structure and functioning can affect crop growth under abiotic stresses, harnessing cultivar-specific microbiome knowledge to enhance crop resilience to stress remains a challenge. Therefore, revealing the distinct variations in compositions and functions of microbes in root-associated niches with different crop species or different cultivars within a species is of great importance for utilizing crop microbiomes to improve performance.

With the increasing demand for agricultural land [12, 13], cadmium (Cd) contamination of farmlands and its accumulation in crops is becoming a serious global environmental issue [14]. Rice is one of the most important staple foods for human beings. Due to the high mobility of Cd from root to shoot in rice, consumption of Cd-contaminated rice products poses a serious threat to public health [15]. In order to decrease the accumulation of Cd in rice grains, apart from applying soil amendments for *in-situ* chemical immobilization, other measures have been widely employed, such as cultivating rice varieties with low Cd accumulation [16]. Enhancing root-associated microbial resistance to Cd stress could also reduce the accumulation of Cd in rice grains, i.e., microbial communities could express functions under elevated Cd levels such as biofilm formation, extracellular polymeric substances (EPS) production, and heavy metal resistance genes and pathways development [17]. Different rice varieties have different capacities for Cd uptake and accumulation [18, 19]. Cao et al. [20] revealed that hybrid rice could accumulate a higher Cd concentration in grains compared with conventional rice, and similar findings were also observed in two major types of Asian cultivated rice, *indica* and *japonica* rice [14]. However, these studies focused on the variations in Cd accumulations of different rice cultivars. Contrasting results might be due to diverse biotic and abiotic factors, such as soil amendments and fertilizer management. Recently, the importance of root-associated microbiomes of different rice cultivars in nutrient cycling and soil organic pollutants degradation have been widely investigated [21, 22], however, there is still a lack of information on root-associated microbial community diversity and community diversity functions of different rice cultivars that exhibit contrasting Cd accumulation traits.

When planting rice in Cd-contaminated soils, substantial evidence suggests that the patterns of root-associated microbial community structures and functions significantly vary between low-Cd rice cultivars and hybrid rice cultivars. Plant-beneficial-taxa such as *Bacillus*, *Pseudomonas*, *Dyella*, and *Rhizobium* are highly enriched in the rhizosphere of low-Cd rice cultivars [23]. These keystone taxa play an essential role in plant growth and Cd resistance in the rhizosphere [24, 25]. Their microbial community assembly is largely governed by interactions among microbes, plant host, and soil environment [26]. Recent insights into assembly patterns in the soil-root continuum can advance our understanding of microbial community response to environmental change [27, 28]. Thus, exploring the formation and maintenance of community assembly is crucial to predicting microbial community variation in taxonomy and functioning [29]. Nevertheless, community assembly in the soil-root continuum and the turnover of microbial functions under Cd stress is not completely understood.

Recently, high-throughput sequencing has been widely used to study the microbiomes of different crops in response to environmental stresses [30, 31]. Metagenomic binning can describe the taxonomic composition and functional potential of microbial communities with high taxonomic resolution [32]. This approach is of great interest for researchers, as it can provide novel insights into the specific ecological adaptation mechanisms and metabolic functions of the microbiota that play important roles in stress tolerance, such as climate change [8], tailing pollution [33, 34], and soil pathogen invasion [35, 36]. Despite this, microbiome data is more diverse and heterogeneous than other types of data, such as soil physicochemical properties. Recently, machine learning has emerged as an effective tool in genomics research since it can provide deeper insights into high-dimensional and sparse microbiome data [37, 38]. Statnikov et al. [39] systematically compared 18 major machine learning classification methods using human microbiome datasets and found that random forest was the most effective machine learning method. At present, machine learning approaches are also widely applied to detect microbial community composition and functional correlation analysis [40]. For instance, Zhang et al. [1] used the random forest approach to predict the root microbiota of *indica* and *japonica* varieties accurately and identified 18 families as biomarker microbes. Also, Yuan et al. [41] accurately predicted the occurrence of soil *Fusarium* wilt using machine learning approaches based on a global integrated bacterial and fungal metadata. Thus, rapid developments in DNA sequencing technology together with enriched publicly available data and emerging machine learning approaches provide a promising approach for researchers to merge data from multiple studies to achieve more robust conclusion.

**Fig. 1:**
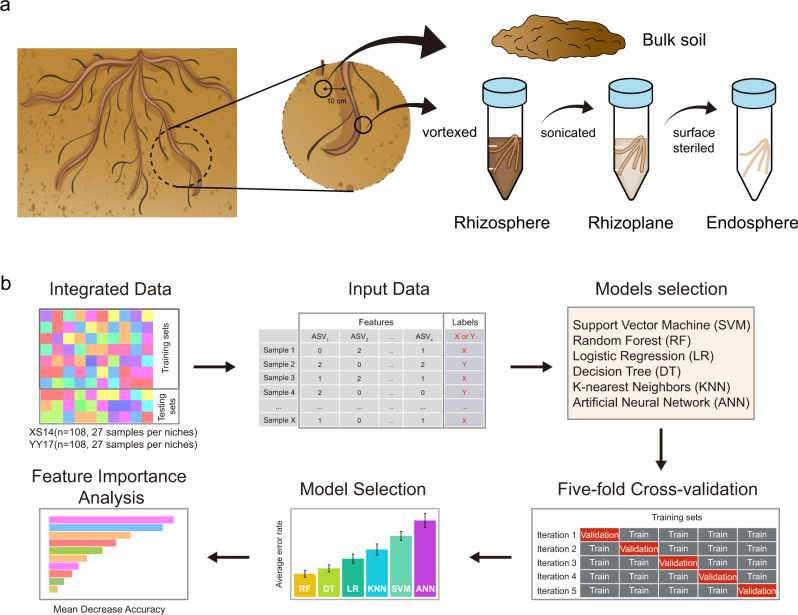
Diagram of sample collection and machine learning model construction. Method of sampling and collecting the microbes in soil and root-associated compartments (**a**) and workflow for implementation of machine learning models for predicting two rice cultivars (**b**).

Previously, we investigated variations in Cd concentrations of different rice cultivars and screened one cultivar with remarkably low Cd accumulation (Xiushui14, XS14) from 10 tested cultivars (Fig. S1). The results in different Cd levels suggested that XS14 exhibited lower Cd accumulation in roots and shoots when compared with YY17 under diverse soil amendment treatments [42]. Keeping these ideas in mind, we aimed to unravel differences in root-associated microbiome assembly and functional attributes of two rice cultivars (XS14 & YY17). We hypothesized that there is a distinct microbial taxon-specific mechanism underpinning the differences in Cd resistance mechanisms between two rice cultivars. To achieve this, we investigated the bacterial community in the soil-root continuum (Fig. 1a) and performed machine learning techniques to identify biomarker taxa of two rice cultivars (Fig. 1b). We also assembled shotgun metagenome to explore the microbiome functional profiles in the rhizosphere and endosphere. This work has developed not only a descriptive understanding of root-associated microbiota but also revealed taxon-specific adaptation mechanisms of two rice cultivars under Cd stress based on metagenomic sequencing analysis and machine learning methods.

## Results

### Cd accumulation features consistently differed between two rice cultivars with varied soil properties

In this study, we used four different soil amendments (lime, pig-manure, biochar, and commercial soil conditioner) to determine whether Cd accumulation features consistently varied between two rice cultivars under different edaphic properties. The application of soil amendments significantly affected soil physicochemical properties (e.g., soil pH, nitrate nitrogen, available Cd) of two rice cultivars (Tables S1, S2). For instance, applications with different amendments also significant effects on the NH_4_^+^-N concentrations in rhizosphere soil of YY17 (*F*_4,10_ = 6.38, *P* < 0.01) and XS14 (*F*_4,10_ = 3.59, *P* < 0.05). As expected, in comparison with YY17, the amount of Cd concentrations in grains reduced significantly in XS14 (Fig. S2b), specifically in treatments with biochar (BC, Student’s *t* test: *t* = 5.401, *df* = 3.983, *P* < 0.01), commercial Mg-Ca-Si conditioner (CMC, Student’s *t* test: *t* = 3.191, *df* = 3.925, *P* < 0.05), and the control (CK, Student’s *t* test: *t* = 3.058, *df* = 3.469, *P* < 0.05). Meanwhile, pH values in bulk and rhizosphere soils of XS14 were remarkably higher than those of the YY17 (e.g., LM in bulk soil, Student’s *t* test: *t* = 3.027, *df* = 3.392, *P* < 0.01; Fig. S2c; Table S2), and XS14 had lower available Cd concentrations in bulk and rhizosphere soils (e.g., CMC in bulk soil, Student’s *t* test: *t* = 2.785, *df* = 2.069, *P* < 0.05; PM in rhizosphere soil, Student’s *t* test: *t* = 4.932, *df* = 3.207, *P* < 0.05; Fig. S2d; Table S2). Most importantly, we found the bioaccumulation factor of YY17 significantly higher than XS14 (Student’s *t* test: *t* = 2.859, *df* = 20.25, *P* < 0.01, Fig. S3). This solid evidence showed that XS14 is a low-Cd rice cultivar compared with YY17. Collectively, our data demonstrate that amendment applications led to the corresponding changes in edaphic properties planted with two rice cultivars, and more importantly, comparisons between two rice cultivars showed that XS14 exerted the lower Cd-accumulating capacity under all types of soil amendments treatments.

### Bacterial diversity and community assembly in four niches of two rice cultivars

**Fig. 2:**
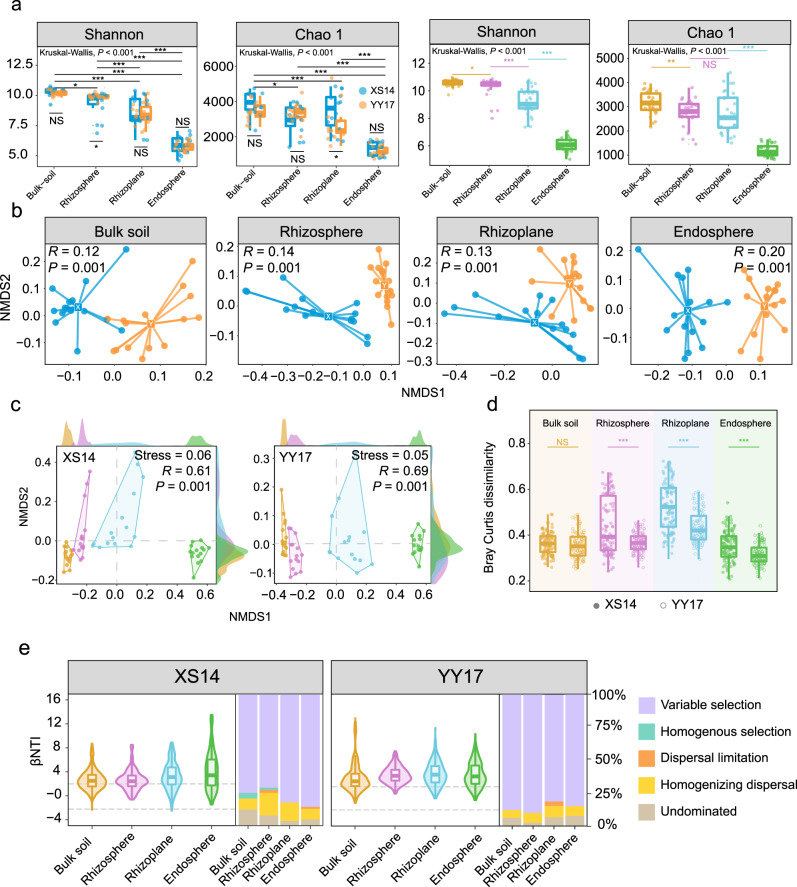
Diversity and assembly of bacterial community. **a** Shannon and Chao1 index of the bacterial community in the four root-associated niches of two rice cultivars. The significance of difference was determined by Kruskal-Wallis test. **b** NMDS analysis grouped by four compartment niches based on Bray-Curtis distance matrices of bacterial communities of two rice cultivars. **c** Microbial Bray-Curtis dissimilarity among all samples varied across four niches between two rice cultivars. In panels (**b**, **c**), *R* and *P* values were evaluated via adonis function in vegan package. **d** Community dissimilarity in four niches between the low-Cd cultivar XS14 and hybrid cultivar YY17. In panels (**a**, **d**), asterisks labeled implies the significant difference (*P* < 0.05) determined by Student’s *t* tests. ‘NS’ represents no significant differences. **e** Deterministic and stochastic processes in bacterial assembly of two rice cultivars among four niches.

To characterize the variations in root-associated microbiota assembly processes of two rice cultivars, we analyzed the 16 S sequencing data from four root-associated niches. After filtering, 11,231,141 high-quality reads were obtained, and these reads were clustered into 14,369 ASVs based on the DADA2 clustering method. For alpha diversity of two kinds of rice, there were no overall differences in the Shannon diversity and Chao 1 index of bulk and endosphere bacterial community between XS14 and YY17. However, Shannon diversity metrics revealed significant higher values in the rhizosphere of YY17 (Student’s *t* test: *t* = 1.975, *df* = 15.034, *P* < 0.05; Fig. 2a), compared with XS14, and Chao 1 index showed the opposite trend in rhizoplane (Student’s *t* test: *t* = 2.011, *df* = 27.79, *P* < 0.05, Fig. 2a). Remarkably, from bulk soil to endosphere, our results showed a gradual decrease along the soil-root continuum whether for Shannon or Chao 1 index (Kruskal-Wallis test, *P* < 0.001; Fig. 2a).

Non-metric multidimensional scaling (NMDS) based on Bray-Curtis distance showed that the bacterial microbiota formed two distinct clusters according to the cultivars among four niches, with the explanation of rice cultivars to bacterial community differences among four niches being 12, 14, 13, and 20%, respectively (Fig. 2b). Additionally, bacterial microbiota of two rice cultivars formed four distinct clusters according to the root niches (Fig. 2c). PERMANOVA analysis suggested that the compartment niches (*R*^*2*^ = 0.59) also strongly affected the microbial community compositions of all samples, and strongly affected XS14 (*R*^*2*^ = 0.61) and YY17 (*R*^*2*^ = 0.69) individually (Fig. 2c; Table S3). The curves of frequency densities showed that samples of four niches were mainly differentiated by the first NMDS axes for two rice cultivars (Fig. 2c). Furthermore, microbial community dissimilarity among all samples of XS14 was much higher than that of YY17 across different niches, except for bulk soil (Student’s t test, bulk soil: *P* = 0.197; rhizosphere, rhizoplane, endosphere: *P* < 0.001, Fig. 2d). In terms of community composition, the dominant phylum among four niches of two rice cultivars was Proteobacteria, Chloroflexi, and Acidobacteria, and the relative abundances of taxa under different treatments also varied between two rice cultivars (Fig. S4a). Notably, the differences in the microbial community composition were detectable at the family level (Fig. S4b, c). For two rice cultivars, families belonged to *Xanthobacteraceae* (7.6–32.4%), *Anaerolineaceae* (10.2–23.1%), *HOC36* (3.7–12.5%), and *Bacillaceae* (2.2–9.6%) were the dominant taxa among four niches, respectively (Fig. S4b). In addition, some potential beneficial bacteria were significantly varied between two rice cultivars among four niches (Fig. S4b, c). For instance, *Pseudomonadaceae* was much richer in bulk soil, rhizosphere, and rhizoplane of XS14 (*P* < 0.05), *Bacillaceae* was obviously enriched in rhizoplane of YY17 (*P* < 0.001), while *Rhizobiaceae* was mainly localized in rhizosphere of XS14 (*P* < 0.001).

SourceTracker model showed that two rice cultivars’ bacterial communities were mainly derived from bulk soils and gradually filtered at different root compartment niches (Fig. S5). Bulk soil bacterial communities were the most important sources of rhizosphere bacterial communities for two rice cultivars, with majority contributions of 79.5 and 84.8% in XS14 and YY17, respectively. Rhizoplane selected majority of species from rhizosphere (79.1% in XS14 and 75.8% in YY17) and the minority of species from bulk soil (8.4% in XS14 and 5.3% in YY17). For two rice cultivars, rhizoplane was the main potential source of endosphere (Fig. S5).

Then, community assembly mechanisms were inferred with the null model analysis. Results showed that variable selection belonging to deterministic processes were the critical process driving bacterial community assembly (~78% for XS14 and ~82% for YY17) among four root-associated niches (Fig. 2e). Furthermore, along with the soil-root continuum, a higher relative contribution of variable selection was observed for bacterial community assembly of XS14 (69~78%) and YY17 (77~80%), and exerted a more significant impact on bacterial community assembly of YY17 than XS14. We also noted that stochastic processes of XS14 in rhizosphere (~25%) contributed more than YY17 (~12%). Collectively, these results suggest that XS14 possessed more distinct and variable bacterial communities assemblages than YY17. The stronger contributions of stochastic processes observed in XS14 may indicate a higher capacity of bacterial community of XS14 in adapting to changes in soil conditions.

### Microbial community co-occurrence networks of two rice cultivars

**Fig. 3:**
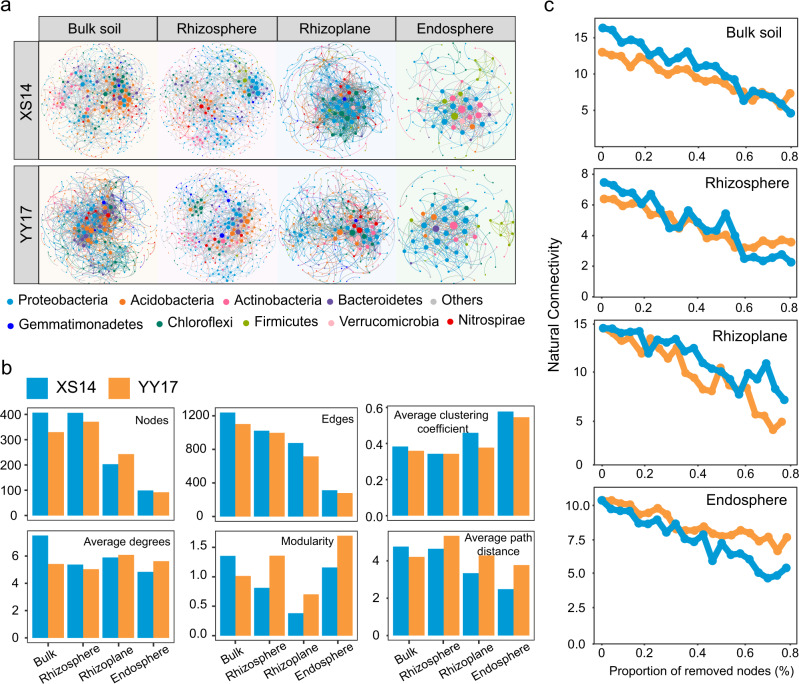
Distinct pattens of the bacterial co-occurrence networks between two rice cultivars. **a** Co-occurrence network analysis of two rice cultivars along the soil-plant continuum. Nodes represent dominant bacterial ASVs (with relative abundances > 0.01%) that were shown in different colors. The node size is proportional to the degree of taxa. **b** Network topological parameters for four root-associated niches bacterial networks. **c** Robustness of bacterial networks in bulk soil, rhizosphere, rhizoplane, and endosphere of XS14 and YY17.

Co-occurrence network analysis based on significant correlations was used to compare the differences of microbial interactions between two rice cultivars across four root niches. The differences in topological properties between empirical networks and random networks suggested that the empirical microbial networks across four niches were not random (Table S4). Consistent with alpha diversity (Fig. 2a), we noted that network complexity gradually decreased, with the highest average degree (7.503 in XS14 and 5.415 in YY17) for networks of bulk soil communities, and lowest average degree (7.503 in XS14 and 5.415 in YY17) for those of endosphere (Fig. 3a, b; Table S5). Co-occurrence network analysis showed that there was a strong host impact on the networks of root-associated bacterial community with a pronounced reduction in the number of nodes and edges of networks from bulk soil to root endosphere (Fig. 3a; Table S5). Additionally, there were more nodes and edges, higher average degree and average clustering coefficient in the networks of XS14 when compared with YY17 (Fig. 3b; Table S5). Further insights into a natural connectivity analysis showed that the XS14 network is much more robust and stable in bulk and rhizosphere soil than that of YY17 (Fig. 3c).

Based on within-module connectivity (Zi) and among-module connectivity (Pi) analysis, we evaluated the topological role of individual nodes in the networks and defined the keystone species in each network of two rice cultivars. Comparison of the module hubs demonstrated that XS14 had more module hubs (nodes highly connected to other members in a module) among four niches than YY17, with a total of 21 and 10 module hubs were detected in XS14 and YY17, respectively (Fig. S6; Table S6). Meanwhile, the number of module hubs decreased from bulk soil (12 and 6 module hubs in XS14 and YY17, respectively) to the endosphere (0 module hubs in XS14 and YY17), and most of the module hubs belonged to the phylum Proteobacteria, Actinobacteria, and Acidobacteria (Table S6). Collectively, our network analysis suggests a more closely connected and stable microbial community in bulk soil and rhizosphere of XS14 when compared with YY17.

### Identifying the core taxonomic community of two rice cultivars using machine learning classification models

**Fig. 4:**
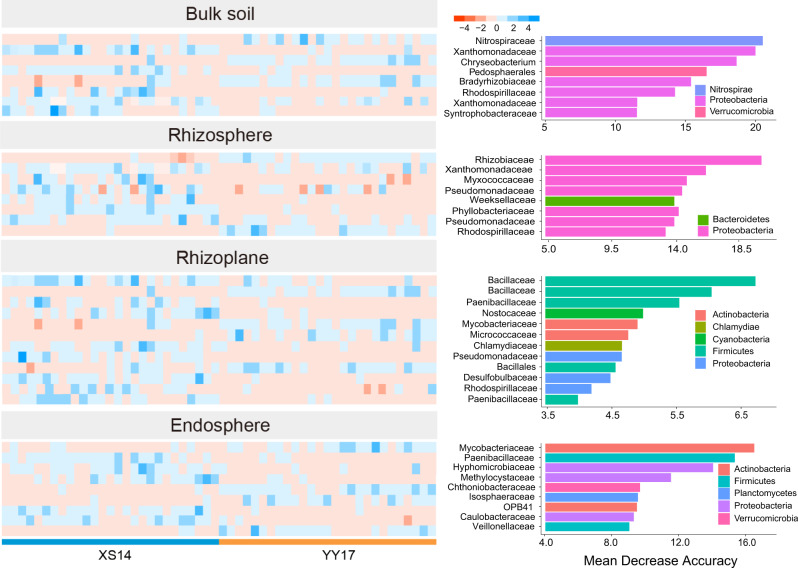
Deciphering indicator biomarkers that predictive for two kinds of rice cultivars. Random forest model used to excavate bacterial species that accurately distinguish samples of XS14 and YY17 among bulk soil, rhizosphere, rhizoplane, and endosphere niches. Top 10 indicator bacterial ASVs in each niche are ranked in descending order of Mean Decrease Accuracy (MDA) values. Heatmap shows the normalized abundances of indicator bacterial ASVs in different treatments. Color represents the phylum of ASVs classified and the taxonomic annotation of ASVs was at family level.

Given the observed dissimilarities in root-associated bacterial communities assemblages between two rice cultivars (Fig. 2), we further explored whether characters of the rice root-associated bacterial communities could be used as biomarker to distinguish two rice cultivars by constructing classification models with machine learning. Relative abundances matrices at the ASVs level among four niches were used as input for model training. We selected six models and performed five-fold cross-validation to verify the models. The best model was then chosen based on average error rates. Our results showed that random forest had the lowest average error rate compared with other models, which is 0.068, 0.067, 0.017, and 0.015 in bulk soil, rhizosphere, rhizoplane, and endosphere, respectively (Fig. S7). Therefore, we further used the random forest model to analyze the importance of each ASV. Confusion matrix showed that the overall prediction accuracy of the random forest model achieved 94.1% (88.9% for XS14 and 100% for YY17), 88.2% (88.9% for XS14 and 87.5% for YY17), 94.1% (100% for XS14 and 90.9% for YY17), and 100% (100% for XS14 and 100% for YY17) in bulk soil, rhizosphere, rhizoplane, and endosphere, respectively (Fig. S8). Cross-validation error curves among four niches showed that the minimum cross-validation error was obtained when using the 8, 8, 12, and 9 most relevant ASVs, respectively (Fig. S9). These biomarkers in four root-associated niches with different relative abundances are predictive of two rice cultivars (Fig. 4). Our result indicated that in bulk soil, *Nitrospiraceae* was the most important ASV and enriched in YY17 samples. *Rhizobiaceae*, *Bacillaceae*, and *Mycobacteriaceae* were the most important ASVs in rhizosphere, rhizoplane, and endosphere, respectively. We further selected the top 2 biomarker ASVs to explore the relationships between relative abundances of keystone biomarker taxa and Cd concentrations in rice grains. Specifically, we found that the relative abundances (Z-score normalized) of the top 2 biomarker taxa among four niches were significantly correlated with Cd concentrations in rice grains (Fig. S10). These results indicated that microbiota colonizing root-associated niches could be used as key biomarkers that made substantial contributions to variations in Cd accumulation capacity of two rice cultivars.

### Specialized distinct functional profiles of rhizosphere and endosphere microbiome between two rice cultivars

**Fig. 5:**
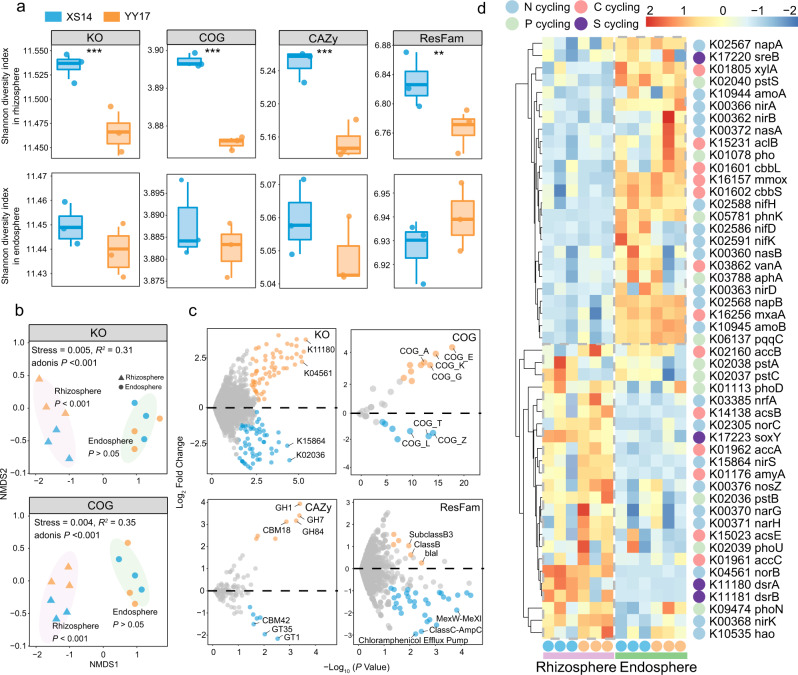
Functional variability in rhizosphere and endosphere microbiome between low-Cd rice XS14 and hybrid rice YY17. **a** Comparison of the functional diversity based on KO, COG, CAZy, and ResFam profiles in rhizosphere and endosphere microbiome. The Kruskal-Wallis test was used to assess the differences, and significant differences are indicated by asterisk (**P < 0.01, ***P < 0.001). **b** NMDS ordinations based on Bray–Curtis distance matrices of KO and COG. The colors and shapes of the symbols indicate the niches and rice cultivars, respectively. Bray-Curtis dissimilarities were tested by PERMANOVA. **c** Volcano plot indicating the differential abundance analysis of microbiome functional profiles based on the KO, COG, CAZy, and ResFam database of XS14 compared with YY17. Several top enriched and depleted functions were labelled in the plots. **d** Heatmap of the relative abundances of the functional genes involved in C, N, P and S cycling based on the KO database. The list of involved genes is provided in Table S8.

After the characterization of bacterial community and identification of biomarker taxa in two rice cultivars, we sought to reveal the potential ecological roles of rhizosphere and endosphere microbiome under Cd stress. We selected 12 DNA samples of the control treatment (CK) from these two compartments for subsequent metagenomic sequencing. Results indicated that microbiome community of XS14 had higher functional diversity (Kruskal-Wallis; Shannon index based on KO: *P* < 0.001, COG: *P* < 0.001, CAZy: *P* < 0.001, and ResFam: *P* < 0.01, Fig. 5a) in the rhizosphere than YY17, however, significant differences were not observed in the endosphere. Functional compositions (NMDS ordinations of KO and COG) of the rhizosphere microbiome community significantly varied from the endosphere, and different rice cultivars also had significant impacts on rhizosphere microbiome functions (PERMANOVA, *P* < 0.001, Fig. 5b), but not in the endosphere. To determine the specialized distinct functional profiles between two rice cultivars, differential enrichment analysis in microbiome genes abundances were performed. Our data revealed that nitric oxide reductase gene (*norB*, *K04561*), sulfite reductase gene (*dsrA*, *K11180*), and amino acid (COG_E) and carbohydrate (COG_G) transport and metabolisms were enriched in XS14 (Fig. 5c). Meanwhile, functional genes involved in C, N, P, and S cycling showed significantly different patterns among niches and rice cultivars (Fig. 5d). For instance, functional genes participated in sulfite reductase (e.g., *dsrA* and *dsrB*) were more abundant in microbiome of the XS14 rhizosphere compared with the YY17, while phosphate transport genes (e.g., *pstA* and *pstC*) were depleted. Compared with the rhizosphere, functional genes involved in carbon (e.g., *cbbL* and *cbbS*) cycling showed an obvious enrichment in endosphere, especially in microbiome of YY17 (Fig. 5d).

**Fig. 6:**
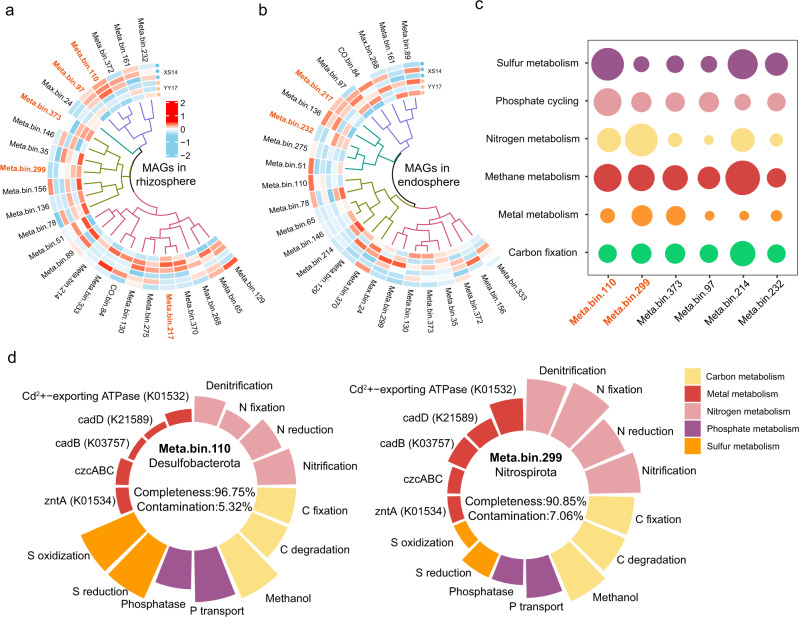
Taxon-specific functions in rhizosphere and endosphere microbiome of two rice cultivars. The distribution of the metagenome-assembled genomes (MAGs) from samples of rhizosphere (**a**) and endosphere (**b**). Orange bold font indicate significantly enriched or depleted MAGs between two rice cultivars. **c** Abundances of genes relating to carbon metabolism, metal metabolism, nitrogen metabolism, phosphate metabolism, and sulfur metabolism from the six MAGs. **d** Genes encoding nitrogen cycling, carbon cycling, phosphorus cycling, sulfur cycling, and metal metabolism in the MAGs affiliated with Nitrospirota and Desulfobacteria. Height of the bar indicates the gene abundance. Different functions categories were shown in different colors.

To provide deeper insights into microbial community member-wide functional capacity of two rice cultivars, we then retrieved the 25 high-quality bacterial MAGs from metagenomic binning (Table S7). Results illustrated different distribution patterns of MAGs in rhizosphere and endosphere of two rice cultivars. The abundances of five of these MAGs (Meta.bin 110, 97, 373, 299, and 217) were significantly different in the rhizosphere, while other two of them (Meta.bin 217 and 232) were remarkably different in the endosphere (Fig. 6a). It is notable that sulfur metabolism genes were more enriched in Meta.bin.110 and nitrogen metabolism genes were more abundant in Meta.bin.299, which belonged to the genus *DP-20* (phylum Desulfobacteria) and *Nitrospira* (phylum Nitrospirota), respectively (Fig. 6b). The Meta.bin.299 was comprised of 3871 contigs with a total length of 3.7 Mb and the Meta.bin.110 was comprised of 5630 contigs with a total length of 5.8 Mb. A more detailed genomic analysis of the two MAGs showed that they had great potential in nutrients cycling and heavy metal metabolism (Fig. 6c). The Meta.bin.110 possessed many elements cycling characteristics, such as sulfur metabolism (e.g., *soxY*, *dsrA*, *dsrB*, *sreB*) and phosphorus metabolism (e.g., *phoD*, *pstA*, *pstC*, *phoN*, and *phoU*) (Fig. 6c; Table S8). The Meta.bin.299 had multiple plant-beneficial genes, such as nitrogen metabolism (e.g., *narG*, *nirB*, *nosZ*, *nirS*, and *norC*) and heavy metal metabolism (e.g., *czcABC* and *Cd*^*2+*^*-exporting ATPase*) (Fig. 6c; Table S8). Also, it should be noted that the Meta.bin.110 and Meta.bin.299 showed different distribution in the rhizosphere of two rice cultivars.

## Discussion

**Fig. 7:**
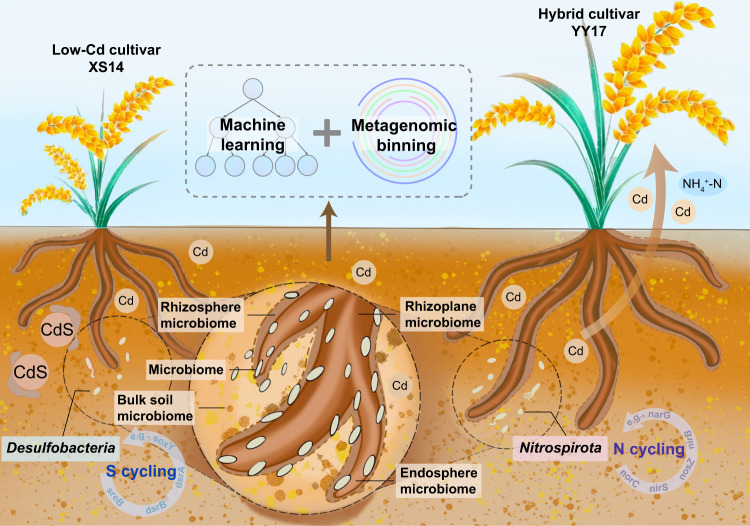
Machine learning and metagenomic analysis jointly identified the biomarker microbes. The conceptual diagram illustrating the microbial taxon-specific survival strategies of XS14 and YY17 in Cd-contaminated soils.

Cd contamination in farmland significantly influenced the soil microbial diversity and food safety [43]. Recently, developing rice cultivars with low Cd accumulation has become a key measure for maintaining food safety under Cd-contaminated soils [44]. Our motivation was to identify keystone taxa that participated in Cd tolerance and nutrient cycling, and improve the understanding of the microbiome-specific mechanisms of low Cd accumulation. In this study, the root-associated microbiome of two rice cultivars representing contrasting Cd accumulation capacities was compared (low-Cd-accumulating cultivar XS14 and hybrid rice cultivar YY17 that selected based on 6-year field experiment). Here, we provide solid evidence that bacterial communities in the soil-root continuum of two rice cultivars had distinct characteristics and reveal the adaptation mechanisms for how the microbiome in rhizosphere and endosphere might have helped rice to grow under Cd stress (Fig. 7).

### Cultivar-specific community assembly and functioning in rice root-associated microhabitats

Understanding the diversity and functional profiles of microbial communities along the soil-root continuum niches is crucial in illustrating microbial assembly mechanisms and their ecological importance under Cd stress. In this study, we characterized the bacterial community diversity and assembly across four root-associated niches of two rice cultivars, and the functional profiles of microbiome in rhizosphere and endosphere. In agreement with previous reports [27, 45], amplicon sequencing data suggested that the root-compartment niches and amendment applications strongly influenced bacterial community diversity and structures, which is confirmed by a declining alpha diversity from bulk soil to endosphere (Fig. 2a). We observed that bacterial community structure (beta diversity) of two rice cultivars significantly varied among four niches (Fig. 2b), which are consistent with the previous finding observations that different rice cultivars harbor distinct microbiota colonizing the root-associated niches [46]. Beta diversity represented the dissimilarity of the community and had strong associations with microbial functions [47]. Particularly, we found that bacterial community of XS14 processed relatively to a higher dissimilarity among four niches compared with YY17 except for the bulk soil (Fig. 2d), and a higher functional diversity was also observed in microbiome of XS14 (Fig. 5a). Based on the above results, we concluded XS14 possessed a more complex and multi-functional microbial community in root-associated niches. Comparison between XS14 and YY17 in network topological parameters indicated that the network of XS14 is much more complex and closely connected than YY17 in bulk and rhizosphere (Fig. 3c). Taken together, our results showed distinct microbial co-occurrence patterns among two cultivars to cope with Cd stress. This is supported by a recent study, reporting that the Pb-accumulating (AE) and non-Pb-accumulating ecotype (NAE) of *S.alfredii* showed distinct recruitment of bacterial communities in rhizosphere with AE forming a more stable community and NAE forming a highly specialized but vulnerable community [48].

Understanding the microbial community assembly mechanisms is essential to advancing microbial ecology research under heavy metal pollution [26]. In our study, deterministic processes (variable selection) dominated the assembly processes of two rice cultivars and gradually increased along the soil-root continuum (Fig. 2e). The fact that the microbiota along continuous root-associated niches is mainly influenced by deterministic processes has been demonstrated in previous studies [6, 27]. As generally accepted, from soils to rhizoplane to endosphere, host selection stress sequentially increased and shaped the bacterial communities [11]. Specifically, our results indicated a stronger signature of stochastic processes (dispersal limitation and homogenizing dispersal) in XS14 compared with YY17 (Fig. 2e). The stronger stochasticity in microbial community assembly indicates that the microbes are less sensitive to variations in edaphic conditions [49] and more adaptable to changed environment [50], evidenced by the higher degree of functional diversification in the root-associated microbiome of XS14. Thus, the bacterial community of XS14 is likely more adapted to Cd-contaminated soil and plays more critical roles in maintaining rice growth under Cd stress.

Soil is a key “seed bank” for the crop microbiome and contains a broad diversity of microbiota [51]. We confirmed that the soil habitats are the major sources of bacterial community among four root-associated niches of two rice cultivars (Fig. S5). Additionally, significant variations in bacterial community assemblages were observed across four root-associated niches between two rice cultivars, with some species within the family *Xanthobacteraceae*, *Bacillaceae*, *Pseudomonadaceae*, and *Rhizobiaceae* (Fig. S4c). The members within *Rhizobiaceae* and *Xanthobacteraceae* are identified as plant growth-promoting bacteria in rhizosphere [52, 53]. The family *Bacillaceae* and *Pseudomonadaceae* can promote nutrient assimilation and enhance crop resistance to environmental perturbation by producing amino acids [24, 54]. Consistently, our metagenomic analyses indicated that functional genes related to amino acid and carbohydrate transport and metabolisms were enriched in XS14 compared with YY17. The amino acid plays a vital role in the chelation of Cd, and carbohydrates are energy storage substances for organism activities that could be significantly upregulated under heavy metal stress [55, 56]. Prior work has suggested that roots could enhance amino acid and carbohydrates metabolisms for alleviating Cd toxicity [57]. Taken together, these results may support the notion that two rice cultivars have distinct function-specific enrichment strategies in microbial community to cope with Cd stress.

### Characterization of the keystone indicator taxa and their diverse ecological functions contributing to distinct survival strategies of two rice cultivars

Recently, network analysis and machine learning are actively applied in microbiome research for identifying the keystone taxa or functions in microbial community [41]. Here, we separately constructed networks in four root-associated niches and performed six machine learning models to explore the keystone indicator taxa of two rice cultivars. Network analysis indicated that the module hubs of two rice cultivars identified by *Zi-Pi* method mainly belonged to the phylum Proteobacteria, Actinobacteria, and Actinobacteria. Meanwhile, higher numbers of nodes and edges and more module hubs in networks of XS14 suggested more complex microbial co-occurrences in root-associated niches of XS14, comparing with YY17 [58]. Due to the extremely high dimensionality of microbiome data, different models showed great variations in prediction accuracy. Random Forest is a supervised machine-learning technique and is suitable for tackling high dimensional and heterogeneous microbiome data [59]. Given that microbial community among root-associated niches could be affected by many factors, such as agronomic practices, climate changes, and host genotypes, thus, we merged our previous study data [60] for the purpose to increase the amount of data and making the results more representative. In this study, the importance analysis realized by the trained Random Forest revealed distinct community signatures among four niches of two rice cultivars. Intriguingly, both the random forest classification model and co-occurrence network analysis indicated that the taxa *Nitrospiraceae*, *Xanthomonadaceae*, and *Mycobacteriaceae* (Fig. 4; Table S6) were the keystone indicator taxa that are predictive for Cd accumulation capacity of two rice cultivars. As mentioned above, these keystone taxa played diverse ecological roles in root-associated niches under Cd stress.

In this study, we further investigated the potential taxon-specific Cd resistance mechanisms underlying diverse Cd accumulation capacity of two rice cultivars in a high taxonomic resolution manner based on metagenomic binning strategy. Notably, we detected two MAGs classified into genus *DP-20* (phylum Desulfobacteria) and *Nitrospira* (phylum Nitrospirota) were significantly more abundant in XS14 and YY17, respectively. Further analysis of genome properties indicated that functions related to sulfur metabolism and nitrogen metabolism were enriched in these two MAGs, respectively. Same with previous studies [61], bins affiliated with Nitrospirota contained high abundance of genes encoding biomolecules (or enzymes) for nitrogen cycling. This suggests potentially increased nitrogen availability with Cd contamination, which has been reported previously [34]. Previous studies also demonstrated that species belonged to Nitrospirota were associated with nitrogen metabolisms and had specific heavy metal resistance mechanisms such as siderophore production, superoxide elimination, and heavy metal efflux [62]. Moreover, some important nitrogen-fixation taxa may contribute to phytoremediation by providing nutrients for hyperaccumulator plants, and more accumulation could be observed in above-ground tissues [34]. This might explain why the root-associated niches of YY17 were colonized by more nitrogen cycling-associated bacteria, so as that more Cd accumulated in grains (Fig. S11). Meanwhile, we showed that the Desulfobacteria*-*affiliated bin contained more sulfur oxidization and reduction genes. Desulfobacteria shows preference for anoxic conditions and is known to be related to iron metabolism and sulfate reduction processes [63]. Indeed, these sulfate-reducing bacteria (SRB) can produce sulfide and immobilize toxic ions like Cd as CdS precipitation, and thus, reduce the bioavailability of Cd in soil [64]. Additionally, given that metal metabolism genes were all detected in two MAGs, it is likely that metal metabolism evolved in microbiota may support both XS14 and YY17 survival in Cd-contaminated soil.

Collectively, the findings of this study enhance our understanding of the distinct microbial taxon-specific survival strategies of two rice cultivars under Cd stress, by identifying keystone biomarkers’ ecological functions. The recovered MAGs provided deep informative knowledge of the variations in functional profiles related to plant growth and heavy metal metabolism of two rice cultivars. However, the trade-offs in cooperation-competition among these keystone taxa are not fully understood and needed to be investigated *in vitro* and *in vivo* for further designing synthetic communities for sustainable agriculture and food security under heavy metal contamination.

## Conclusions

Our work investigated structural and functional features of root-associated microbiomes of two rice cultivars with contrasting Cd accumulation capacities (low-Cd rice XS14 and hybrid rice YY17), and revealed distinct taxon-specific and function-specific recruitment strategies in root-associated niches between these two cultivars. We identified biomarker members belonging to *DP-20* (phylum Desulfobacteria) and *Nitrospira* (phylum Nitrospirota) which showed significant but contrasting ecological functioning in sustaining the survival adaptabilities of XS14 and YY17, respectively, through sulfur metabolism in *DP-20*, and nitrogen and metal metabolism in *Nitrospira*. Our study methodically characterizes the variations in Cd accumulation in grains of two rice cultivars to decipher taxonomy-function-specific microbial recruitment mechanisms. Our findings highlight cultivar-specific patterns of microbial community assembly and functioning in the soil-root continuum of two rice cultivars in response to Cd stress, and lay the foundation for potential manipulation of biomarkers to promote rice growth and food safety in Cd-contaminated soils.

## Materials and methods

### Field experiment description and sampling

The field trial was carried out in Wenling county (28°21′ N, 121°15′ E), Zhejiang province (southeast China) with a subtropical monsoon climate. The sampling sites and details have been described in our previous study [60]. We conducted a 6-year consecutive field experiment with the application of diverse soil amendments with two rice cultivars since 2016, one is a hybrid rice (*Oryza sativa* L., Japonica) named Yongyou17 (YY17), the other is a conventional rice (*Oryza sativa* L., Japonica) named Xiushui14 (XS14). Based on our previous studies results (Fig. S1), in the subsequent analysis, we defined XS14 as the low-Cd rice cultivar and YY17 as the control cultivar. The experimental site consisted of five treatments (control, CK; lime, LM; biochar, BC; pig manure, PM; commercial soil conditioner, CMC) with a completed randomized block design (5 × 4 m for each plot) of three replicates (Fig. S2a). The detailed amendment scheme and fertilization management have been described in a previous study [60].

Soil and root samples collection were performed in rice harvested stage (20th October 2020), corresponding to four root-associated niches including bulk soil, rhizosphere, rhizoplane, and endosphere, respectively. Bulk soil samples were collected from a distance of 10 cm away from the rice roots within each treatment plot by thoroughly mixing five random topsoil cores (0–20 cm), and five random rice roots were collected and mixed to form one root sample at the same time. The root-associated samples collection and compartments processing were performed as previously described [4, 60]. All soil and rice root samples were stored at −80 °C prior to DNA extraction (Fig. 1a). A total of 120 DNA samples (2 rice cultivars × 5 treatments × 3 replicated × 4 compartments) were obtained for microbial community analysis. The physicochemical properties of soil samples were measured as previously reported [60], including soil pH, dissolved organic nitrogen (DON), dissolved organic carbon (DOC), total nitrogen (TN), total carbon (TC), available phosphorus (AP), nitrate nitrogen (NO_3_^-^-N), ammonium nitrogen (NH_4_^+^-N), available Cd (CaCl_2_ extractable Cd) and total Cd concentrations in rice grains and bulk soil of two rice cultivars. The bioaccumulation factors of two cultivars were calculated as Cd concentrations in rice grains / total Cd concentrations in bulk soils [42].

### DNA extraction and bacterial 16 S rRNA gene amplicon sequence processing

All the genomic DNA was extracted using FastDNA SPIN kit for soil (QBIO gene Inc., Carlsbad, CA, USA) following the manufacturer’s instructions. The bulk and rhizosphere soil DNA were extracted from 0.5 g fresh soil, the rhizoplane soil DNA was extracted from 0.2 g fresh soil, and the endosphere sample DNA was extracted from 3–5 g fresh roots. The analyses for bacterial community among four niches were conducted via 16 S rRNA gene amplicon sequencing. The V4 region was amplified using the common primer pairs 515 F (5′-GTGCCAGCMGCCGCGGTAA-3′) and 806 R (5′-GGACTACHVGGGT-WTCTAAT-3′) [31]. All DNA samples were sequenced on the Illumina Nova6000 platform from Guangdong Magigene Biotechnology Co. Ltd. (Guangzhou, China). Primer sequences with a quality score (Q) below 30 were trimmed and pair-end sequences were joined by using Quantitative Insight into Microbial Ecology2 (QIIME2 v. 2020.11) pipeline [65]. High-quality sequencing data were categorized into amplicon sequence variants (ASVs) using DADA2 pipeline and taxonomic assignment was performed using the Silva database (v13.2) (https://www.arb-silva.de/). Before subsequent analysis, the ASVs table was rarefied to the lowest number of sequences across all samples.

### Metagenomic sequencing and data analysis

To further characterize the microbiome ecological functions of two rice cultivars, 12 DNA samples of the control treatment collected from two rice cultivars (CK treatment × two niches × three replicates × two cultivars) were selected for metagenomic sequencing using the Illumina NovaSeq PE150 platform (Personal Biotechnology Co., Ltd. Shanghai, China). In total, 240 Gb of raw data were obtained with approximately 20 Gb per sample. Raw reads were subjected to quality control and filtering using FastQC (v0.11.9) and Trimmomatic (v0.39) [66], respectively. Then, reads were mapped to the rice reference genome (*Oryza sativa* L., GCF_000005425.2), and mapped reads were removed using Bowtie2 [67]. The remaining clean data were assembled into contigs using Megahit (v1.2.9, parameter: –min-contig-len 500 –k-step 20) [68]. In total, we retrieved 19,512,653 contigs with an average GC content is 63.43%, N50 is 1011 bp, and a total length is 8.356 × 10^9 ^bp. All the contigs greater than 1000 bp were used for further gene annotation using Prodigal (v2.6.3, parameter: -p meta) [69], and generated 5,327,157 Open Reading Frames (ORFs) with 45.1% of the genes were identified as complete. Then, ORFs were clustered at 0.95 similarity threshold using CD-HIT (v4.8.1, parameter: -aS 0.9 -c 0.95 -G 0 -g 0 -T 0 -M 0) [70] to generate non-redundant gene catalog. The abundance profiles of genes were estimated in transcripts per million (TPM) by salmon (v1.3.0, parameter: –meta) [71]. The Kyoto Encyclopedia of Genes and Genomes (KEGG) Orthology (KO) profiles [72], Carbohydrate Active Enzyme (CAZyome) [73], and Clusters of Orthologous Groups of proteins (COG) [74] database were used for microbiome functional annotation using eggNOG (v5.0) [75]. The antibiotic resistance genes were also detected using the ResFams database [76]. Functional diversities were calculated using R software. Differential functional abundance analysis between XS14 and YY17 was performed using generalized linear model (GLM) approach (TMM normalization method) in *edgeR* (v3.28.1) package [77].

Genome binning was performed using CONCOCT (v1.0.0), MaxBin (v2.2.6), MetaBAT (v2.12.1) by MetaWRAP pipeline (v1.3.2) [78]. DAS_Tool (v1.1.2, parameter: –search_engine diamond) [79] was further used to select the best metagenome-assembled genomes (MAGs) from the three binning software results. dRep (v3.0.0, parameter: -sa 0.99 -pa 0.95 -nc 0.3) [80] was used for dereplication across all bins at the threshold of 99% ANI with at least 30% overlap between genomes. CheckM (v1.1.3) [81] was used to assess the completeness and contamination of the retrieved MAGs. Finally, we obtained 67 MAGs that meet the threshold of medium-quality (>50% completeness and <10% contamination) and 19 MAGs that were estimated to be high-quality genome (>90 completeness and <10% contamination) [8]. Then, only 25 MAGs with the completeness >80% and contamination <10% were selected for further analysis [33]. The abundances of the MAGs across the samples were estimated using MetaWRAP (v1.3.2) [78] with the *quant_bins* module. Taxonomy of the MAGs was assigned using GTDB-TK (V1.7.0) [82]. ORFs were predicted using Prodigal (v2.6.3) [69] and functional annotation was performed by KofamKOALA [83] against the KEGG database.

### Machine learning algorithms implementation and selection

We performed six machine learning approaches for classification, including artificial neural network (ANN), decision tree (DT), random forest (RF), K-nearest neighbors (KNN), logistic regression (LR), and support vector machine (SVM) with radial kernel. Bacterial 16 S rRNA amplicon sequencing data from our previous study [60] were integrated to increase the amount of training data, including the similar four amendments treatments with the same rice cultivars (XS14 and YY17). In total, 216 bacterial samples were collected with 54 samples per niches. The dataset was divided into two partitions, one is the training set (70%), and the other is the testing set (30%), with the former used for training models, while the latter was used for testing their predictive abilities. Five-fold cross-validation was employed to avoid overfitting Briefly, the training set was randomly separated into five subsets, one of them was used for validation and the remaining were used for training, and the process was repeated for five iterations. The average error rates of each model were calculated to select the optimal model that can give the most accurate predictions. The flowchart of the machine learning classification models is illustrated in Fig. 1b. The construction of data preprocessing and model prediction was performed in *R* software.

### Statistical analysis

We calculated microbial alpha diversity based on the rarefied ASVs table using the *vegan* package [84]. ANOVA was performed using Tukey’s HSD test to assess the statistical significance of multiple comparisons. Kruskal-Wallis test was used to compare the significance of differences in alpha diversity among four niches. Student’s *t* tests (two-sided) were used to compare the differences in two rice cultivars. Microbial beta diversity was evaluated by calculating Bray-Curtis distance matrices and non-metric multidimensional scaling (NMDS) analyses were used for ordination. Permutational multivariate analyses of variance (PERMANOVA) based on Bray-Curtis distance matrices were performed with the *adonis* function to assess the effects of different factors on microbial communities. We also used the Source Tracker model [85] to estimate the potential sources of bacterial communities in the four niches. Differential enrichment analysis in microbiome genes abundances was performed using *R* package *edgeR*. Only genes with FDR-adjusted *P* < 0.05 and log2 (fold change) >1 or < −1 were regarded as significantly “enriched” or “depleted”, respectively [86].

Beta Nearest Taxon Index (βNTI) was calculated using the null model (999 randomizations) to evaluate the relative importance of determinism and stochasticity in bacterial community assembly processes [87]. Furthermore, a Bray-Curtis-based Raup–Crick matrix (RC_bray_) was calculated to partition processes as: (1) homogeneous selection (βNTI > 2), (2) variable selection (βNTI < −2), (3) dispersal limitation (|βNTI | <2 and RC_bray_ > 0.95), (4) homogenizing dispersal (|βNTI | <2 and RC_bray_ < −0.95), and (5) undominated (|βNTI | <2 and |RC_bray_ | < 0.95) [87].

The network analysis was performed based on a significant Spearman correlation matrix and visualized using the *Gephi* platform (version 0.9.2) [88] based on the Fruchterman Reingold algorithm. To eliminate the influence of the rare ASVs in the networks, we focused on the ASVs that were detected in at least 75% samples and with relative abundance >0.01% [16]. Only the robust (r > 0.7 or r < −0.7) and statistically significant (FDR adjust *P* < 0.05) [89] correlations were kept. Topological characteristics were calculated using *igraph* package [90]. Random networks with the same edges and nodes were generated, and topological properties of random networks are calculated based on Erdős–Rényi model [91]. Natural connectivity was calculated to reveal the robustness of networks according to the previous study [92]. Briefly, nodes were sorted by betweenness and then we sequentially removed until 80% of the nodes were removed, and then evaluate the robustness of the network after deleting nodes [93]. We also calculated the within-module connectivity (*Zi*) and among-module connectivity (*Pi*) according to the method previously described [94]. Module hubs (Zi > 2.5, Pi ≤ 0.62) are highly connected nodes within modules, which were expected to mediate interactions between species within modules [58]. Therefore, in this study, module hubs are regarded as key nodes. All statistical analysis and graph plotting were produced in *R* program.

## Supplementary information

Revised Supplementary Information

## Data Availability

The raw sequencing data have been submitted in the Genome Sequence Archive in the BIG Data Center, Chinese Academy of Sciences (http://bigd.big.ac.cn/gsa) under accession number CRA006325 (16 S) and CRA006366 (metagenomic).
