# Supplementary material for: Metagenomic and machine learning-aided identification of biomarkers driving distinctive Cd accumulation features in the root-associated microbiome of two rice cultivars
Source: ISME Commun. 2023 Feb 22;3:14. doi: 10.1038/s43705-023-00213-z (PMC9947119; doi:10.1038/s43705-023-00213-z)
Supplement: Supplementary file 1 — Revised Supplementary Information [file 43705_2023_213_MOESM1_ESM.docx]

**Supplementary figures**

**Fig. S1** Two typical rice cultivars screened from on our previous field studies. **a** Cd concentration in rice grains of 10 cultivars. **b** The bioaccumulation factors of 10 cultivars. Bars with different letters indicate significant differences as defined by Tukey’s HSD test (*P* < 0.05).

**Fig. S2** Field experiment design and variations in soil properties and Cd concentrations in grains. **a** Schematic diagram of the field experiment design. **b** Cd accumulations (total Cd concentrations) in rice grains under different soil amendments treatments of two kinds of rice. **c** Changes of pH values in bulk and rhizosphere soil under different soil amendments treatments of two kinds of rice. **d** Changes of available (CaCl_2-_extracted) Cd concentrations in bulk soil and rhizosphere soil under different soil amendments treatments of two kinds of rice. Asterisks indicate significant differences as defined by the Student’s t test (*indicates *P* < 0.05, **indicates *P* < 0.01).

**Fig. S3** The bioaccumulation factors of XS14 and YY17. Student’s t test was used to analyze the significant differences between the cultivars.

**Fig. S4** Variations in microbial taxonomic community structure of two rice cultivars. **a** Relative abundance of dominant bacterial phyla of XS14 and YY17 root-associated microbiota among four compartment niches under different soil amendment treatments. LM: lime; BC: biochar; PM: pig manure; CMC: commercial Mg-Ca-Si conditioner. **b** Different dominant bacterial family of XS14 and YY17among four niches. Low abundance family with relative abundances less than top ten of the total sequences across all samples are grouped into “Low abundance”. **c** Significant difference analysis for relative abundances of dominant family between XS14 and YY17 among four niches. Different colors indicate significant differences as defined by student’s test as shown in the legend. Letters in each plot indicate significant higher relative abundance in XS14 (X) or YY17 (Y).

**Fig. S5** SourceTracker model showed the potential sources of bacterial communities of XS14 and YY17 in each compartment niche. U: unknown source.

**Fig. S6** The keystone species are identified based on the node topological roles in networks of XS14 and YY17 among four compartment niches. The topological role of each node is determined according to the scatter plot of within-module connectivity (*Zi*) and among-module connectivity (*Pi*). A node belonged to a module hub was identified if its Zi ≥ 2.5. The module hubs in different niches represented by different colors.

**Fig. S7** The average error rate of five-fold cross-validation based on six machine learning algorithms in each compartment niche. Error bars represent standard deviations. Different letters above bars indicate significant differences (*P* < 0.05).

**Fig. S8** Performance of the random forest classification model. **a** Samples (54 samples per niche and 216 samples in total) used in random forest analysis. The data set was divided into two parts, including training set (70% of the data set) and testing set (30% of the data set). **b** Confusion matrix indicated the performance of random forest model on the testing data.

 **Fig. S9** The cross-validation error curves of random forest model across four niches. The top 8, 8, 12, and 9 biomarker ASVs identified by RF classification of two rice cultivars samples.

**Fig. S10** The linear relationships between normalized relative abundances of top 2 keystone biomarker ASVs among four niches and normalized Cd concentrations in rice grains. P values were indicated by asterisks: **P* < 0.05, ***P* < 0.01, and ****P* < 0.001.

**Fig. S11** The concentrations of nitrate nitrogen and ammonium nitrogen in XX14 and YY17 soil samples. Significant differences were defined by the student’s t test.

**Supplementary tables**

**Table S1.** The properties of bulk and rhizosphere soils of two rice cultivars in rice harvested stage.

| Samples | Treatments | NO_3_^-^-N  (mg kg^-1^) | NH_4_^+^-N  (mg kg^-1^) | AP  (mg kg^-1^) | DOC  (mg kg^-1^) | DON  (mg kg^-1^) | TC  (g kg^-1^) | TN  (g kg^-1^) |
| --- | --- | --- | --- | --- | --- | --- | --- | --- |
| XS14 | | | | | | | | |
| Bulk soil | CK | 13.7±1.2a | 10.4±1.7a | 7.5±0.9a | 72.3±4.96bc | 17.3±6.1a | 2.55±0.16b | 0.27±0.04a |
|  | LM | 12.4±2.4a | 11.0±0.9a | 22.9±19.9a | 73.3±11.9bc | 16.1±6.8a | 2.16±0.34bc | 0.22±0.04a |
|  | BC | 14.7±1.6a | 11.6±1.4a | 17.6±9.1a | 44.6±1.5c | 18.1±1.8a | 2.99±0.16a | 0.27±0.02a |
|  | PM | 13.5±2.2a | 12.4±0.6a | 13.9±0.4a | 84.0±24.3ab | 12.7±1.7a | 2.56±0.15b | 0.26±0.02a |
|  | CMC | 13.6±3.3a | 12.5±2.5a | 11.9±4.9a | 111.1±24.7a | 13.9±3.0a | 2.10±0.24c | 0.22±0.03a |
|  |  |  |  |  |  |  |  |  |
| Rhizosphere | CK | 10.0±0.8b | 9.5±0.6b | 13.3±7.8a | 52.6±9.4b | 13.9±1.0ab | 2.87±0.10ab | 0.24±0.01a |
|  | LM | 12.0±0.8ab | 12.3±2.2a | 16.1±6.2a | 48.5±7.6b | 12.3±3.2ab | 2.41±0.04b | 0.24±0.01a |
|  | BC | 11.9±2.5ab | 9.7±0.4b | 21.4±13.5a | 71.1±42.0ab | 9.8±1.4b | 3.00±2.00a | 0.28±0.09a |
|  | PM | 13.5±1.2a | 9.6±0.7b | 17.5±6.9a | 107.6±36.2a | 16.5±3.1a | 2.71±0.16ab | 0.24±0.02a |
|  | CMC | 13.8±0.7a | 9.4±0.7b | 18.6±14.9a | 67.8±14.2ab | 12.3±4.9ab | 2.53±0.29ab | 0.25±0.03a |
| YY17 | | | | | | | | |
|  | CK | 13.3±1.7b | 11.1±1.8a | 11.2±4.9a | 81.2±60.7a | 13.3±6.1a | 2.87±0.19ab | 0.26±0.01a |
|  | LM | 15.1±3.7ab | 13.2±1.7a | 16.1±9.3a | 90.9±22.8a | 16.9±7.71a | 2.39±0.26b | 0.24±0.02a |
| Bulk soil | BC | 18.2±2.1a | 13.2±1.7a | 11.7±5.9a | 53.4±5.9a | 10.9±3.4a | 2.98±0.26a | 0.25±0.03a |
|  | PM | 15.5±2.4ab | 11.7±1.1a | 8.9±1.9a | 121.4±34.7a | 22.8±6.8a | 2.72±0.29ab | 0.27±0.03a |
|  | CMC | 16.6±1.9ab | 14.0±3.1a | 8.9±2.9a | 76.8±36.2a | 12.7±5.9a | 2.54±0.31ab | 0.28±0.03a |
|  |  |  |  |  |  |  |  |  |
| Rhizosphere | CK | 11.1±2.2b | 16.0±0.5a | 9.1±6.5a | 28.6±19.8b | 8.9±3.4b | 2.27±0.19a | 0.22±0.20a |
|  | LM | 11.3±0.6b | 17.0±1.1a | 6.7±4.4a | 23.4±10.9b | 10.7±2.4b | 2.37±0.05a | 0.23±0.01a |
|  | BC | 12.6±0.9ab | 13.4±4.1ab | 14.6±7.2a | 54.7±24.8ab | 14.2±6.9ab | 2.54±0.11a | 0.23±0.01a |
|  | PM | 15.2±1.8a | 11.4±1.3b | 11.5±3.1a | 83.2±32.2a | 23.1±6.2a | 2.48±0.72a | 0.24±0.07a |
|  | CMC | 14.5±0.2a | 9.9±1.0b | 6.2±3.5a | 57.9±41.4ab | 16.2±3.9ab | 2.38±0.21a | 0.23±0.03a |

Abbreviations: CK: Control; LM: Lime; BC: Biochar; PM: Pig-manure; CMC: commercial Mg-Ca-Si conditioner. Values are mean ± SD of three replications; values within a column followed by different letters mean significant differences in bulk soils or rhizosphere soils (*P* < 0.05).

**Table S2.** The pH and available Cd concentrations in bulk and rhizosphere soil and total Cd concentrations in bulk soil in two rice cultivars in rice harvested stage.

| Treatment ^a^ | pH^B^ | pH^R^ | ACd^B^ | ACd^R^ | TCd |
| --- | --- | --- | --- | --- | --- |
| XS14 | | | | | |
| CK | 6.17±0.08c | 5.88±0.13b | 0.30±0.07ab | 0.22±0.09a | 0.72±0.32a |
| LM | 6.43±0.05a | 5.92±0.11b | 0.28±0.21ab | 0.27±0.05a | 0.92±0.56a |
| BC | 6.39±0.04ab | 6.19±0.09a | 0.22±0.07b | 0.24±0.03a | 0.56±0.04a |
| PM | 6.20±0.08c | 5.88±0.07b | 0.46±0.09a | 0.31±0.09a | 0.73±0.20a |
| CMC | 6.43±0.06a | 6.04±0.10ab | 0.21±0.02b | 0.26±0.05a | 0.82±0.25a |
| YY17 | | | | | |
| CK | 6.08±0.05b | 5.77±0.10c | 0.47±0.14a | 0.35±0.10ab | 0.53±0.18a |
| LM | 6.18±0.03b | 5.84±0.17bc | 0.50±0.18ab | 0.31±0.10b | 0.60±0.18a |
| BC | 6.34±0.11a | 6.10±0.11a | 0.32±0.11ab | 0.28±0.04b | 0.58±0.29a |
| PM | 6.13±0.10b | 5.86±0.10bc | 0.5±0.13b | 0.47±0.07a | 0.53±0.09a |
| CMC | 6.30±0.08a | 6.06±0.12ab | 0.47±0.16ab | 0.33±0.07ab | 0.51±0.24a |

^a^ Abbreviations: CK: Control; LM: Lime; BC: Biochar; PM: Pig-manure; CMC: commercial Mg-Ca-Si conditioner. pH^B^: bulk soil pH; pH^R^: rhizosphere soil pH; ACd^B^: available Cd concentrations in bulk soil; ACd^R^: available Cd concentrations in rhizosphere soil.

^b^ The data means mean ± SD. Different letters mean p <0.05.

**Table S3.** Effects of compartment niche, rice type and soil amendments treatments on the microbial community base on PERMANOVA.

|  | Comp | Type | Treat | Comp × Type | Comp × Treat | Type× Treat | Comp × Type × Treat |
| --- | --- | --- | --- | --- | --- | --- | --- |
| All samples | | | | | | | |
| *F* value | 146.43 | 17.77 | 4.73 | 6.12 | 1.74 | 2.24 | 1.31 |
| *R^2^* (%) | 0.59 | 0.02 | 0.05 | 0.02 | 0.06 | 0.02 | 0.04 |
| *P* value | 0.001*** | 0.001*** | 0.001*** | 0.001*** | 0.001*** | 0.01** | 0.05* |
| XS14 | | | | | | | |
| *F* value | 37.2 | / | 3.0 | / | 1.7 | / | / |
| *R^2^* (%) | 0.61 | / | 0.1 | / | 0.1 | / | / |
| *P* value | 0.001*** | / | 0.01** | / | 0.02* | / | / |
| YY17 | | | | | | | |
| *F* value | 48.1 | / | 2.9 | / | 1.2 | / | / |
| *R^2^* (%) | 0.69 | / | 0.1 | / | 0.1 | / | / |
| *P* value | 0.001*** | / | 0.01** | / | 0.21 | / | / |

^a^ *P* value was tested based on 999 permutations.

^b^ Abbreviations: Comp: Compartment; Treat: Treatment

**Table S4.** Topological features of the co-occurrence networks of two rice cultivars of random data

|  | Topological parameter | Bulk soil | Rhizosphere | Rhizoplane | Endosphere |
| --- | --- | --- | --- | --- | --- |
| XS14 | Node | 407 | 406 | 203 | 99 |
|  | Edge | 1238 | 1022 | 875 | 313 |
|  | Average degree | 6.086±0.000 | 5.032±0.000 | 8.621±0.000 | 6.322±0.000 |
|  | Modularity  Average clustering coefficient  Average path distance | 0.328±0.012  0.015±0.002  3.524±0.010 | 0.372±0.013  0.012±0.003  3.875±0.017 | 0.267±0.012  0.042±0.004  2.673±0.007 | 0.311±0.019  0.064±0.009  2.673±0.017 |
| YY17 | Node | 330 | 371 | 243 | 92 |
|  | Edge | 1102 | 997 | 716 | 280 |
|  | Average degree | 6.682±0.000 | 5.375±0.000 | 5.894±0.000 | 6.087±0.000 |
|  | Modularity  Average clustering coefficient  Average path distance | 0.314±0.012  0.020±0.003  3.361±0.009 | 0.359±0.013  0.015±0.003  3.692±0.015 | 0.344±0.015  0.024±0.004  3.218±0.013 | 0.323±0.019  0.067±0.011  2.680±0.019 |

**Table S5.** Topological features of the co-occurrence networks of two rice cultivars among four compartment niches

| Topological parameter | Node | Edge | Average degree | Modularity | Average clustering coefficient | Average path distance |
| --- | --- | --- | --- | --- | --- | --- |
| XS14 | | | | | | |
| Bulk soil | 407 | 1238 | 7.503 | 1.356 | 0.382 | 4.763 |
| Rhizosphere | 406 | 1022 | 5.375 | 0.812 | 0.343 | 4.645 |
| Rhizoplane | 203 | 875 | 5.893 | 0.382 | 0.459 | 3.342 |
| Endosphere | 99 | 313 | 4.923 | 1.159 | 0.581 | 2.485 |
| YY17 | | | | | | |
| Bulk soil | 330 | 1102 | 5.415 | 1.015 | 0.357 | 4.212 |
| Rhizosphere | 371 | 997 | 5.034 | 1.359 | 0.343 | 5.338 |
| Rhizoplane | 243 | 716 | 6.087 | 0.702 | 0.377 | 4.284 |
| Endosphere | 92 | 280 | 5.621 | 1.698 | 0.547 | 3.774 |

| Niches | Phylum | Family | Degree | Zi | Pi | Module ID |
| --- | --- | --- | --- | --- | --- | --- |
| XS14 | | | | | | |
| Bulk soil | Actinobacteria | unassigned | 29 | 3.08 | 0.19 | 4 |
|  | Nitrospirota | *Nitrospiraceae* | 9 | 2.55 | 0.38 | 3 |
|  | Chloroflexi | *Caldilineaceae* | 16 | 2.50 | 0.12 | 2 |
|  | Proteobacteria | *Nitrosomonadaceae* | 20 | 2.94 | 0.19 | 1 |
|  | Actinobacteria | unassigned | 26 | 2.92 | 0.08 | 4 |
|  | Bacteroidota | *BSV26* | 5 | 2.53 | 0.36 | 5 |
|  | Desulfobacterota | unassigned | 22 | 3.38 | 0.17 | 1 |
|  | Proteobacteria | *Gallionellaceae* | 24 | 3.67 | 0.30 | 2 |
|  | Acidobacteria | *Vicinamibacteraceae* | 17 | 2.51 | 0.11 | 1 |
|  | Actinobacteria | *Mycobacteriaceae* | 19 | 3.20 | 0.10 | 2 |
|  | Proteobacteria | *Xanthomonadaceae* | 17 | 2.50 | 0.22 | 2 |
|  | Chloroflexi | *Anaerolineaceae* | 22 | 3.38 | 0.17 | 1 |
| Rhizosphere | Desulfobacteria | unassigned | 15 | 2.75 | 0.44 | 4 |
|  | Proteobacteria | *Nitrosomonadaceae* | 29 | 3.39 | 0.07 | 5 |
|  | Proteobacteria | *Gallionellaceae* | 25 | 2.76 | 0.08 | 5 |
|  | Proteobacteria | *Xanthomonadaceae* | 14 | 2.97 | 0.26 | 3 |
|  | Proteobacteria | *HOC36* | 29 | 3.39 | 0.07 | 5 |
| Rhizoplane | Acidobacteria | *Acidobacteriaceae* | 19 | 2.81 | 0.29 | 2 |
|  | Acidobacteria | *Mycobacteriaceae* | 11 | 2.66 | 0.32 | 4 |
|  | Firmicutes | *Bacillaceae* | 30 | 2.81 | 0.58 | 2 |
|  | Proteobacteria | *Sphingomonadaceae* | 20 | 3.05 | 0.27 | 2 |
| Endosphere | / | / | / | / | / | / |
| YY17 | | | | | | |
| Bulk soil | Proteobacteria | *Bradyrhizobiaceae* | 9 | 3.36 | 0.21 | 3 |
|  | Myxococcota | *Haliangiaceae* | 20 | 3.17 | 0.10 | 2 |
|  | Bacteroidota | *Chitinophagaceae* | 38 | 2.65 | 0.45 | 1 |
|  | Chloroflexi | *Anaerolineaceae* | 19 | 2.95 | 0.10 | 2 |
|  | Chloroflexi | *Anaerolineaceae* | 11 | 3.18 | 0.52 | 4 |
|  | Acidobacteria | *Chitinophagaceae* | 26 | 3.17 | 0.44 | 2 |
| Rhizosphere | Acidobacteria | unassigned | 26 | 2.78 | 0.15 | 2 |
|  | Sva0485 | *Haliangiaceae* | 28 | 2.61 | 0.32 | 2 |
|  | Proteobacteria | *Myxococcaceae* | 11 | 2.91 | 0.17 | 1 |
|  | Proteobacteria | *Caulobacteraceae* | 17 | 2.91 | 0.59 | 1 |
|  | Chloroflexi | *Anaerolineaceae* | 21 | 2.91 | 0.26 | 4 |
| Rhizoplane | / | / | / | / | / | / |
| Endosphere | / | / | / | / | / | / |

**Table S6** The nodes identified as module hubs in microbial co-occurrence networks on four niches between two cultivars

**Table S7** Information of the metagenome assembled genomes (MAGs) from metagenome of rhizosphere and endosphere of two kinds of rice.

|  | Phylum | Completeness  (%) | Contamination  (%) | N50 | GC |
| --- | --- | --- | --- | --- | --- |
| CO.bin.84 | Proteobacteria | 94.56 | 3.1 | 13079 | 64.98 |
| Max.bin.24 | Actinobacteria | 98.99 | 3.3 | 152374 | 70.9 |
| Max.bin.268 | Bacteroidetes | 84.91 | 2.9 | 15543 | 38.52 |
| Meta.bin.110 | Desulfobacterota | 96.75 | 5.32 | 21721 | 57.89 |
| Meta.bin.129 | Proteobacteria | 80.64 | 4.54 | 11515 | 66.8 |
| Meta.bin.130 | Actinobacteria | 88.14 | 6.12 | 20956 | 67.91 |
| Meta.bin.136 | Bdellovibrionota | 90.7 | 0.84 | 44879 | 52.95 |
| Meta.bin.146 | Actinobacteria | 93.68 | 9.4 | 12298 | 53.34 |
| Meta.bin.156 | Actinobacteria | 97.14 | 3.37 | 14156 | 71.22 |
| Meta.bin.161 | Nitrospirota | 94.03 | 5.51 | 12588 | 58.6 |
| Meta.bin.214 | Proteobacteria | 91.56 | 5.17 | 20904 | 64.87 |
| Meta.bin.217 | Firmicutes | 97.32 | 0.67 | 36545 | 41.3 |
| Meta.bin.232 | Proteobacteria | 94.15 | 0.97 | 85411 | 37.26 |
| Meta.bin.275 | Chlamydia | 84.59 | 1.38 | 22786 | 40.18 |
| Meta.bin.299 | Nitrospirota | 90.85 | 7.06 | 11451 | 55.82 |
| Meta.bin.333 | Proteobacteria | 92.89 | 5.24 | 22443 | 63.27 |
| Meta.bin.35 | Bacteroidetes | 90.18 | 3.91 | 10488 | 37.6 |
| Meta.bin.370 | Actinobacteria | 83.35 | 4.28 | 14930 | 73.45 |
| Meta.bin.372 | Myxococcota | 93.87 | 5.16 | 13617 | 69.17 |
| Meta.bin.373 | Actinobacteria | 94.78 | 9.02 | 20166 | 57.64 |
| Meta.bin.51 | Bacteroidetes | 97.29 | 2.71 | 26373 | 44.02 |
| Meta.bin.65 | Actinobacteria | 93.53 | 4.7 | 9642 | 75.52 |
| Meta.bin.78 | Gemmatimonadota | 80.48 | 8.79 | 10490 | 66.23 |
| Meta.bin.89 | Proteobacteria | 90.99 | 6.83 | 11137 | 60.66 |
| Meta.bin.97 | Proteobacteria | 98.85 | 2.59 | 17784 | 39.62 |

**Table S8** Information of the carbon fixation, methane metabolism, nitrogen metabolism, phosphate cycling, and sulfur metabolism genes.

| Function |  | Gene | KID |
| --- | --- | --- | --- |
| Carbon metabolism | C fixation | *cbbL* | K01601 |
|  |  | *cbbS* | K01602 |
|  |  | *aclB* | K15231 |
|  |  | *accA* | K01962 |
|  |  | *accB* | K02160 |
|  |  | *accC* | K01961 |
|  |  | *acsB* | K14138 |
|  |  | *acsE* | K15023 |
|  | C degradation | *xylA* | K01805 |
|  |  | *amyA* | K01176 |
|  |  | *vanA* | K03862 |
|  | Methanol | *mmox* | K16157 |
|  |  | *mxaA* | K16256 |
| Nitrogen metabolism | N fixation | *nifD* | K02586 |
|  |  | *nifH* | K02588 |
|  |  | *nifK* | K02591 |
|  | N reduction | *nasB* | K00360 |
|  |  | *nasA* | K00372 |
|  |  | *nirD* | K00363 |
|  |  | *nirA* | K00366 |
|  |  | *nirB* | K00362 |
|  |  | *narG* | K00370 |
|  |  | *narH* | K00371 |
|  |  | *nrfA* | K03385 |
|  |  | *napA* | K02567 |
|  |  | *napB* | K02568 |
|  | Nitrification | *amoA* | K10944 |
|  |  | *hao* | K10535 |
|  |  | *amoB* | K10945 |
|  | Denitrification | *norB* | K04561 |
|  |  | *nosZ* | K00376 |
|  |  | *nirS* | K15864 |
|  |  | *nirK* | K00368 |
|  |  | *norC* | K02305 |
| Phosphate cycling | P transport | *pstA* | K02038 |
|  |  | *pstB* | K02036 |
|  |  | *pstC* | K02037 |
|  |  | *pstS* | K02040 |
|  |  | *phnK* | K05781 |
|  | Phosphatase | *phoD* | K01113 |
|  |  | *phoN* | K09474 |
|  |  | *phoU* | K02039 |
|  |  | *aphA* | K03788 |
|  |  | *pqqC* | K06137 |
|  |  | *pho* | K01078 |
| Sulfur metabolism | S oxidization | *soxY* | K17223 |
|  | S reduction | *dsrA* | K11180 |
|  |  | *dsrB* | K11181 |
|  |  | *sreB* | K17220 |
